# Effect of Iron Deficiency on Right Ventricular Strain in Patients Diagnosed with Acute Heart Failure †

**DOI:** 10.3390/jcm14155188

**Published:** 2025-07-22

**Authors:** Kemal Engin, Umit Yasar Sinan, Sukru Arslan, Mehmet Serdar Kucukoglu

**Affiliations:** Department of Cardiology, Istanbul University-Cerrahpasa Cardiology Institute, 34098 Istanbul, Turkey; enginnkemal@gmail.com (K.E.); sukru.arslan@iuc.edu.tr (S.A.); kucukoglu3@yahoo.com (M.S.K.)

**Keywords:** iron deficiency, right ventricular strain, acute heart failure, echocardiography, RV-GLS, subclinical dysfunction

## Abstract

**Background:** Iron deficiency (ID) is a prevalent comorbidity of heart failure (HF), affecting up to 59% of patients, regardless of the presence of anaemia. Although its negative impact on left ventricular (LV) function is well documented, its effect on right ventricular (RV) function remains unclear. This study assessed the effects of ID on RV global longitudinal strain (RV-GLS) in patients diagnosed with acute decompensated HF (ADHF). **Methods:** This study included data from 100 patients hospitalised with ADHF irrespective of LV ejection fraction (LVEF) value. ID was defined according to the European Society of Cardiology HF guidelines as serum ferritin <100 ng/mL or ferritin 100–299 ng/mL, with transferrin saturation <20%. Anaemia was defined according to World Health Organization criteria as haemoglobin level <12 g/dL in women and <13 g/dL in men. RV systolic function was assessed using parameters including RV ejection fraction (RVEF), tricuspid annular plane systolic excursion (TAPSE), RV fractional area change (FAC), peak systolic tissue Doppler velocity of the RV annulus (RV TDI S′), acceleration time of the RV outflow tract, and RV free wall GLS. **Results:** The mean (±SD) age of the study population (64% male) was 70 ± 10 years. The median LVEF was 35%, with 66% of patients classified with HF with reduced ejection fraction, 6% with HF with mid-range ejection fraction, and 28% with HF with preserved ejection fraction. Fifty-eight percent of patients had ID. There were no significant differences between patients with and without ID regarding demographics, LVEF, RV FAC, RV TDI S′, or systolic pulmonary artery pressure. However, TAPSE (15.6 versus [vs.] 17.2 mm; *p* = 0.05) and RV free wall GLS (−14.7% vs. −18.2%; *p* = 0.005) were significantly lower in patients with ID, indicating subclinical RV systolic dysfunction. **Conclusions:** ID was associated with subclinical impairment of RV systolic function in patients diagnosed with ADHF, as evidenced by reductions in TAPSE and RV-GLS, despite the preservation of conventional RV systolic function parameters. Further research validating these findings and exploring the underlying mechanisms is warranted.

## 1. Introduction

Iron deficiency (ID) is a prevalent comorbidity of heart failure (HF) that significantly worsens clinical outcome(s) and prognosis [1,2]. Studies have demonstrated that ID leads to impaired mitochondrial function and decreased myocyte contractility, highlighting its detrimental effects independent of anaemia in patients diagnosed with HF [3,4]. While most studies investigating ID in HF have focused on stable patients with reduced ejection fraction (EF) [2,5], recent clinical observations have reported ID in up to 80% of patients diagnosed with acute decompensated HF (ADHF) [6]. Moreover, ID was associated with short-term adverse outcomes in ADHF [6,7]. Although the negative impact of ID on left ventricular (LV) systolic function is well established, its effects on right ventricular (RV) function, particularly RV global longitudinal strain (RV-GLS), as an indicator of early subclinical dysfunction, remain insufficiently studied [8,9,10].

Previous studies have demonstrated the adverse effects of ID on RV systolic function in patients diagnosed with ADHF [11]. However, the specific impact of ID on RV-GLS, which reflects early subclinical impairment, has not yet been adequately explored. As such, the present study aimed to evaluate the relationship between ID and RV-GLS in patients diagnosed with ADHF.

## 2. Methods

### 2.1. Study Population

Data from 100 patients hospitalised with ADHF between January 2022 and September 2022 were included in this study. Acute heart failure was diagnosed based on the European Society of Cardiology (ESC) criteria and defined as rapid onset or worsening of HF signs and symptoms requiring intravenous (IV) diuretic therapy or hospitalisation [12].

**Figure 1:**
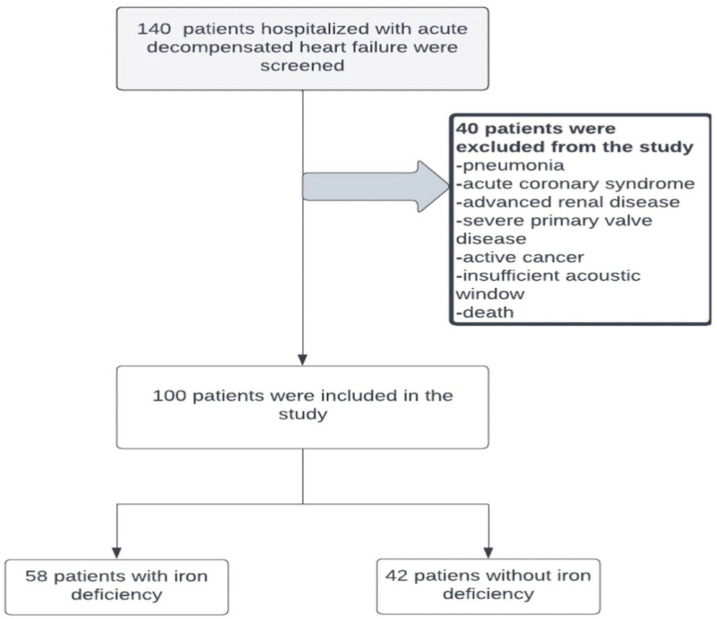
Flow diagram of patient inclusion and exclusion. Of the 140 patients hospitalized with acute decompensated heart failure screened, 40 were excluded due to comorbid conditions or insufficient data. The final study population consisted of 100 patients, of whom 58 had iron deficiency and 42 did not.

Patients with active infections, primary tricuspid valve disease, severe rheumatic mitral stenosis, acute coronary syndrome, pneumonia, advanced kidney or liver failure, pulmonary arterial hypertension, or active cancer, as well as patients who died during hospitalisation, were excluded (Figure 1).

The study protocol adhered to the ethical principles outlined in the Declaration of Helsinki, and informed consent was obtained from all participants before enrolment. Our study was initiated with the approval of the local ethics committee (E-69291215-900-8625).

On admission, demographic data, comorbidities, vital signs, and standard treatments were recorded for each patient. Baseline assessments included 12-lead electrocardiogram and laboratory investigations for renal function and electrolyte, pro-B-type natriuretic peptide (pro-BNP), and troponin levels. Additional laboratory investigations, including complete blood count and ferritin, serum iron, cholesterol, and C-reactive protein levels, were performed approximately 120 h after admission.

Patients were categorised into two groups based on the presence or absence of ID, as defined by the ESC guidelines.

### 2.2. ID Parameters Assessment and Definitions

Blood parameters for diagnosing ID and assessing inflammation were obtained approximately 120 h after admission, following clinical stabilisation. Clinical stabilisation was defined as the absence of the need for IV diuretics, transition to oral diuretic therapy, and no requirement for mechanical support devices while maintaining haemodynamic stability.

The ESC criteria for ID diagnosis were used to define ID as ferritin level < 100 µg/L or ferritin levels between 100 and 299 µg/L with transferrin saturation (TSAT) < 20% [12]. Anaemia is defined by the World Health Organization (WHO) as a haemoglobin level < 12 g/dL in women and <13 g/dL in men. N-terminal proBNP levels were measured on admission. Ferritin, haemoglobin, and complete blood counts were analysed using a laboratory analyser (cobas 6000, Roche Diagnostics, Mannheim, Germany), and NT-proBNP levels were measured using the cobas E411 system (Roche, Germany). TSAT was calculated as serum iron divided by the total iron-binding capacity (TIBC).

Subgroup analyses were performed to compare patients with iron deficiency alone versus those with combined iron deficiency and anaemia, in order to explore potential differential effects on RV function. This stratification was based on clinical considerations, as the presence of anaemia may amplify or modify the impact of iron deficiency.

### 2.3. Echocardiographic Evaluation

Standard two-dimensional (2D) echocardiography (2DE) evaluations were performed approximately 120 ± 24 h after stabilisation using a Epiq7 system (Philips Healthcare, Andover, MA, USA) equipped with an X5 transducer. Echocardiographic images were reviewed by two experienced cardiologists who were blinded to the clinical data.

The measurements followed the American Society of Echocardiography [13] standards and included views from the parasternal long axis, parasternal short axis, apical four-chamber, three-chamber, and two-chamber. The key parameters assessed were as follows:

**Left atrium (LA):** diameter at end systole along the parasternal long axis.
**Pulmonary valve:** Assessed using continuous wave (CW) and pulsed wave (PW) Doppler on the parasternal short axis. Pulmonary acceleration time was measured using PW Doppler across the pulmonary valve.
**LV systolic function:** Calculated using the modified Simpson biplane method by manually tracing the apical four- and two-chamber views.
**RV systolic function:** Evaluated using the apical four-chamber view. The key parameters were as follows:
  - Tricuspid annular plane systolic excursion (TAPSE) measured via M-mode.
  - Peak systolic tricuspid annular velocity (RV TDI S′) was obtained using tissue Doppler imaging.
  - RV fractional area change (RV FAC) was calculated as the percentage of RV end-diastolic and end-systolic area changes.
  - Systolic pulmonary artery pressure was estimated using the peak velocity gradient of tricuspid regurgitation flow, right atrial pressure derived from the inferior vena cava dimensions, and respiratory variability.

Two-dimensional strain imaging was used to assess myocardial deformation as an early marker of RV systolic dysfunction. The time of aortic valve closure was considered end-systole, whereas the R-wave peak on the electrocardiogram was considered end-diastole. For strain measurements, an apical four-chamber view of the RV was captured, ensuring that all RV segments and the LV apex were within the imaging frame [14,15].

Boundaries of the endocardium, myocardium, and epicardium were automatically defined and manually adjusted to avoid errors. Analysis was performed using QLAB-CMQ software (version 10.8) on the Philips Epiq 7C system. The RV free wall was divided into three segments (basal, mid, and apical), and the mean longitudinal strain was calculated across these segments. Patients with incomplete imaging windows or unmeasurable strain values were excluded.

**Figure 2:**
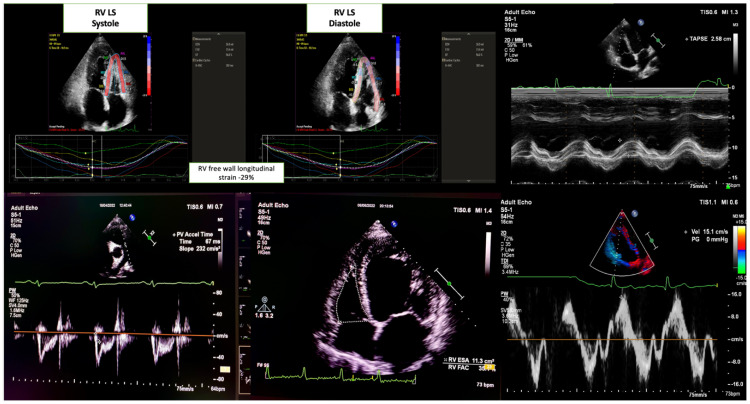
Evaluation of Right ventricular function in patients with acute decompensated heart failure using speckle tracking and other parameters echocardiography.

The parameters used for RV function evaluation in this study are reported in Figure 2 [14] and Supplementary Figure S1.

### 2.4. Statistical Analysis

Statistical analyses were performed using SPSS version 21 (IBM Corp., Armonk, NY, USA). Continuous variables are expressed as mean ± standard deviation (SD), while categorical variables are expressed as percentage. The normality of data distribution was assessed using the Kolmogorov–Smirnov test.

#### Comparisons Between Groups

Continuous variables were tested for normality using the Shapiro–Wilk test. As most continuous variables were not normally distributed, they are presented as median (min–max), and categorical variables are presented as frequencies and percentages. Continuous variables were analysed using the independent samples T-test for normally distributed data or the Mann–Whitney U test for non-normally distributed data. Categorical variables were compared using the chi-squared (χ^2^) test.

The Spearman’s correlation test was used to investigate the relationships between continuous variables. Independent predictors of reductions in RV global longitudinal strain (GLS) reduction were identified using multiple linear regression analysis. The regression model included parameters such as haemoglobin, LVEF, heart rate, deceleration time (DKB), and troponin and ferritin levels.

Differences with *p* < 0.05 were considered to be statistically significant.

## 3. Results

**Figure 3:**
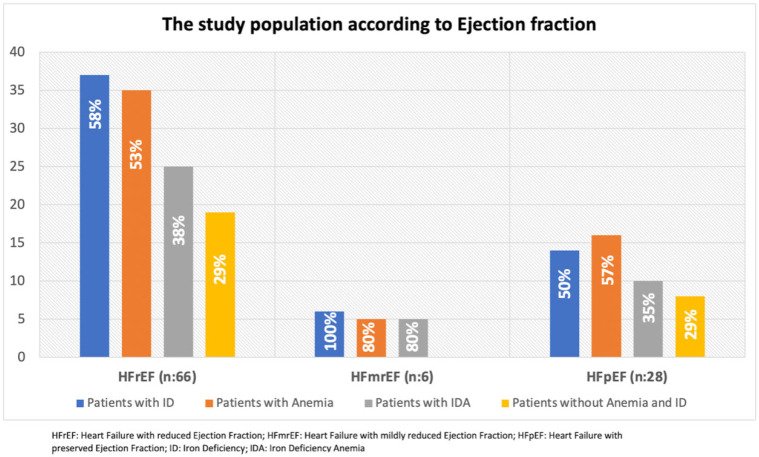
Distribution of the study population according to ejection fraction subgroups and the presence of iron deficiency, anemia, and both conditions combined.

The distribution of patients according to HF classification is illustrated in Figure 3. The majority (66%) of the population consisted of patients with HF with reduced ejection fraction (HFrEF). Additionally, 41% of patients fulfilled the criteria for both anaemia and ID.

Patients were divided into two groups based on the presence or absence of ID. The clinical, laboratory, demographic, and echocardiographic characteristics of the study groups are summarised in Table 1. No significant differences were observed between the groups in terms of age; sex; or comorbidities including diabetes, hypertension, and asthma/chronic obstructive pulmonary disease. Laboratory investigations revealed similar levels of creatinine, sodium, potassium, creatine kinase, cholesterol, C-reactive protein, pro-BNP, and troponin. However, haemoglobin, haematocrit, ferritin, and TSAT levels were significantly lower in the group with ID (*p* < 0.01).

In the echocardiographic assessments, LVEF, LA diameter, FAC, pulmonary artery systolic blood pressure, and tissue Doppler imaging values for the RV were similar between groups. However, RV-GLS was significantly lower in the ID group (*p* = 0.005), while TAPSE and pulmonary acceleration time showed a trend towards lower values in the ID group (*p* = 0.05 for both).

In the subgroup analysis, patients with both ID and anaemia were compared to those with ID alone. The ECHO parameters of the two groups are summarised in Table 2. Although TAPSE and RV-GLS values were numerically lower in the group with both ID and anaemia, the differences were not statistically significant (*p* = 0.08 and *p* = 0.66, respectively). No significant differences were observed in the other echocardiographic parameters.

Another subgroup analysis compared patients with ID only to those without ID or anaemia. The ECHO parameters of the two groups are summarised in Table 2. While TAPSE exhibited no significant difference, RV-GLS tended to be lower in the ID group, reflecting subclinical systolic dysfunction (*p* = 0.05).

Independent predictors of reduced RV-GLS were evaluated using multiple regression analysis. The model included haemoglobin, LVEF, glomerular filtration rate, heart rate, DKB, troponin, ferritin, and LA diameter. The adjusted R^2^ value for the model was 0.23. Haemoglobin levels (B = 0.652, *p* = 0.010) and LVEF (B = 0.158, *p* < 0.0001) were identified as independent predictors of RV-GLS deterioration (Table 3).

Spearman correlation analysis revealed a weak―but statistically significant―relationship between ferritin and RV-GLS, haemoglobin and RV-GLS, and pulmonary acceleration time (Table 4).

## 4. Discussion

The present study investigated the effect of ID on RV systolic function in patients hospitalised for ADHF who underwent transthoracic echocardiography. The main findings are as follows. First, in the ID group, TAPSE (15.6 mm vs. 17.2 mm; *p* = 0.05) and RV-GLS (−14.7% vs. −18.2%; *p* = 0.005) were significantly lower compared with the non-ID group. Second, in patients with ID but no anaemia, the RV-GLS (−20.4% vs. −16.2%, *p* = 0.05) was significantly lower than that in non-ID patients. Third, multiple linear regression analysis revealed that haemoglobin (B = 0.652, *p* = 0.010) and LVEF (B = 0.158, *p* < 0.0001) independently predicted impairment in RV-GLS.

HF is a global health problem that requires frequent hospitalisation and is associated with high mortality and morbidity rates. ID is a common condition in patients with HF, affecting 30–50% of those with chronic HF and up to 70–80% of those with acute HF. Previous studies have demonstrated that ID impairs cardiomyocyte contractile function, and that iron replacement therapy improves this function [16,17]. Studies have also reported significant symptom improvement and better clinical outcomes in patients with HF treated with iron replacement therapy [18,19].

Although the effect of ID on LV systolic and diastolic functions has been well studied, its impact on RV systolic function remains unclear. Previous research has shown that RV systolic dysfunction in patients with HF is associated with poorer outcomes, regardless of LVEF [20,21,22]. RV function is particularly vulnerable to conditions that increase workload and comorbidities, such as ID, which negatively affect systolic and diastolic function [23,24]. In a study involving 903 patients with acute HF, Minana et al. [11] reported that ID was associated with a reduction in TAPSE. However, this study did not find a relationship between ID and LVEF. Similarly, our findings confirmed a significant reduction in TAPSE in the ID group, while no difference in the LVEF was observed. Additionally, our study revealed that RV-GLS was significantly lower in patients with ID, suggesting subclinical myocardial dysfunction.

In our subgroup analysis, patients with ID were compared to those without ID. RV-GLS was lower in the ID group, suggesting that ID is associated with impaired RV myocardial deformation detectable by strain imaging, even when TAPSE remains preserved in the early stages of ID. This supports the higher sensitivity of RV-GLS compared to TAPSE in detecting subclinical dysfunction. Previous studies have compared RV-GLS in patients with and without anaemia but have not specifically focused on those with ID or ID anaemia. For example, Burns et al. [25] investigated echocardiographic parameters in patients with HF, preserved ejection fraction, and anaemia. Their study found reduced FAC and increased systolic pulmonary artery pressure but no differences in LVEF or strain parameters between the groups. Similarly, studies by Opeyemi et al. [26] and Barbosa et al. [27] assessed RV function in patients with sickle cell anaemia and found that, while the FAC was similar between the groups, TAPSE and tissue Doppler imaging values were altered. Our findings are consistent with these studies, further emphasising the unique effect of ID on RV function.

In this study, subgroup stratifications were performed to explore potential differences between iron deficiency alone and combined iron deficiency with anaemia, as these conditions may have distinct pathophysiological impacts on RV function. Observed borderline associations (e.g., TAPSE *p* = 0.05) should be interpreted as trends rather than definitive findings.

Mechanistically, several pathways may explain the observed myocardial dysfunction caused by ID [3,28,29]. Iron plays a critical role in oxygen transport and storage, cellular energy metabolism via the electron transport chain, contractile protein synthesis, and antioxidant defence mechanisms. ID can lead to oxidative stress, impaired contractile function, and myocyte apoptosis. These mechanisms may explain the reduction in RV-GLS observed in this study, even in the absence of significant changes in traditional echocardiographic parameters.

## 5. Limitations

The present study was observational in design; as such, causality between ID and RV dysfunction could not be established. In addition, it is a single-centre design, and the relatively small sample size may limit the generalisability of the results. Future multicentre studies with larger and more diverse cohorts are needed to validate these findings, to better understand the clinical impact of iron deficiency on RV function in AHF, and to explore the effects of iron replacement on RV function and clinical outcomes. In this cohort, intravenous iron therapy was not systematically administered during hospitalisation, and post-discharge treatment decisions were left to the discretion of the primary treating physician. This represents a limitation of the study, and future prospective investigations incorporating iron therapy outcomes would further enhance clinical relevance. Finally, while our regression model identified haemoglobin and LV-EF as significant predictors of RV-GLS, the model demonstrated modest explanatory power (R^2^ = 0.23), suggesting that other factors may also contribute. Given the older age of the study population, additional factors such as comorbidity burden, inflammatory markers, and nutritional status—although not included in the current analysis—may also influence RV function and warrant consideration in future research.

## 6. Conclusions

ID was associated with the subclinical impairment of RV systolic function in patients diagnosed with ADHF. This dysfunction was evident even in the absence of anaemia, highlighting the need for routine assessment of iron status and the potential benefits of iron replacement therapy in improving myocardial function and clinical outcomes.

## Figures and Tables

**Table 1:** Demographic, laboratory, and echocardiographic characteristics of patients with and without iron deficiency.

Variable	Iron Deficiency (−) (n = 42)	Iron Deficiency (+) (n = 58)	*p*-Value
Age (years)	68 (56–85)	70 (54–87)	0.65
Gender (male), n (%)	29 (69.0)	35 (60.3)	0.40
Hypertension, n (%)	33 (78.6)	47 (81.0)	0.80
Diabetes mellitus, n (%)	19 (45.2)	33 (56.9)	0.31
Ischaemic heart disease, n (%)	27 (64.3)	44 (75.9)	0.26
Asthma/COPD, n (%)	4 (9.5)	14 (24.1)	0.07
Diuretic treatment prior to admission, n (%)	40 (95.2)	57 (98.3)	0.57
Heart rate (b.p.m.)	76 (60–120)	84 (55–115)	0.38
SBP (mmHg)	130 (90–160)	127 (100–155)	0.67
Severe TR, n (%)	6 (14.3)	15 (25.9)	0.21
Haemoglobin (g/dL)	13.5 (10.5–16.0)	11.6 (9.1–14.8)	<0.01 *
Haematocrit (%)	40.2 (31.8–49.0)	34.7 (26.4–45.1)	<0.01 *
Serum iron (μg/dL)	50 (34–128)	35 (16–198)	<0.01 *
Ferritin (ng/dL)	156.6 (112–954)	61.9 (10.3–169)	<0.01 *
TSAT (%)	26.3 (17.7–60.1)	14.5 (4.7–78.1)	<0.01 *
WBC (×10^3^/mL)	8.2 (4.5–64)	8.1 (4.4–12)	0.25
CRP (mg/dL)	5.0 (0.4–19)	6.7 (0.6–32.4)	0.23
Creatinine (mg/dL)	1.2 (0.5–1.9)	1.1 (0.6–2.0)	0.98
eGFR (mL/min/1.73 m^2^)	60.1 (30.5–120)	53.4 (32.7–120)	0.44
Serum sodium (mmol/L)	139.2 (130–145)	138.1 (129–145)	0.55
Serum potassium (mmol/L)	4.3 (3.0–5.3)	4.5 (3.2–5.4)	0.56
ALT (U/L)	20.5 (6–46)	17 (3–43)	0.08
NT-proBNP (pg/mL)	4500 (1000–20,000)	3925 (1500–35,000)	0.79
Troponin (ng/mL)	0.021 (0.007–0.05)	0.018 (0.003–0.07)	0.60
Total cholesterol (mg/dL)	142 (72–234)	140 (91–298)	0.68
LDL-cholesterol (mg/dL)	84 (34–156)	75 (39–219)	0.70
HDL-cholesterol (mg/dL)	40 (24–63)	39.5 (21–93)	0.46
Echocardiographic findings			
LVEF (%)	32.5 (20–60)	36 (18–60)	0.91
LAD (mm)	48 (40–66)	46.5 (42–61)	0.59
TAPSE (mm)	17.2 (13–22.8)	15.6 (12–22.9)	0.05
RV FAC (%)	38.3 (24–54)	36.4 (21–53)	0.21
RV-LS (%)	−18.2 (8.6–27.2)	−14.7 (6.3–26.8)	0.005 *
Pulmonary acceleration time (ms)	97.8 (60–140)	88.8 (55–130)	0.05
PASP (mmHg)	45 (30–100)	50 (28–80)	0.32
RV TDI S’ (cm/s)	10.7 (4.2–15.7)	9.4 (5.3–17.0)	0.09

* *p* < 0.05, statistically significant. HTC: haematocrit; TSAT: transferrin saturation; WBC: white blood cell; CRP: C-reactive protein; eGFR: estimated glomerular filtration rate; ALT: alanine transaminase; NT-proBNP: amino-terminal pro-brain natriuretic peptide; LDL: low-density lipoprotein; HDL: high-density lipoprotein; LVEF: left ventricular ejection fraction; LAD: left atrial diameter; TAPSE: tricuspid annular plane systolic excursion; RV FAC: right ventricular fractional area change; RV-LS: right ventricular longitudinal strain; PASP: pulmonary artery systolic pressure; RV TDI S’: right ventricular tissue Doppler imaging systolic wave S’ velocity.

**Table 2:** Echocardiographic findings stratified by iron deficiency (ID) and anaemia status. Comparison groups: (1) anaemia (−), ID (+) vs. anaemia (+), ID (+); (2) anaemia (−), ID (−) vs. anaemia (−), ID (+).

Parameter	Anaemia (−) ID (+) (n = 17)	Anaemia (+) ID (+) (n = 41)	* p * -Value	Anaemia (−) ID (−) (n = 27)	Anaemia (−) ID (+) (n = 17)	* p * -Value
LVEF (%)	35 (18–60)	38 (18–60)	0.41	30 (20–60)	35 (18–60)	0.42
LAD (mm)	49 (44–60)	46 (42–61)	0.12	48 (40–61)	49 (44–60)	0.36
TAPSE (mm)	18 (12–22)	15.2 (12–22.9)	0.08	17.3 (14.1–22.8)	18 (12–22)	0.90
FAC (%)	38.6 (20–50)	36 (20–53)	0.39	38 (18.5–50)	38.6 (20.7–50)	0.09
RV-LS (%)	−16.2 (6.3–26.8)	−14.7 (6.5–25.8)	0.66	−20.4 (−9.9–27)	−16.2 (6.3–26.8)	0.05 *
Pulmonary acceleration time (ms)	92 (50–116)	88 (56–132)	0.91	96.9 ± 22.1	86.9 ± 21.1	0.20
PASP (mmHg)	50 (30–80)	50 (28–70)	0.72	45 (30–100)	50 (30–80)	0.68
RV TDI S’ (cm/s)	10.3 (5.3–16.3)	9.1 (6.4–17)	0.78	12.2 (4.2–15.4)	10.3 (5.3–16.3)	0.21

* *p* < 0.05, statistically significant. LVEF: left ventricular ejection fraction; LAD: left atrial diameter; TAPSE: tricuspid annular plane systolic excursion; FAC: fractional area change; RV-LS: right ventricular longitudinal strain; PASP: pulmonary artery systolic pressure; RV TDI S’: right ventricular tissue Doppler imaging systolic velocity.

**Table 3:** Factors associated with right ventricular longitudinal strain in multiple linear regression analysis.

Variable	Unstandardised Coefficient (B)	Std. Error	Beta	* p * -Value	95% CI
Constant	−0.961	7.507		0.89	−15.872 to 13.950
Haemoglobin (HGB)	0.652	0.248	0.252	0.01 *	0.160 to 1.145
LVEF (%)	0.158	0.035	0.429	<0.01 *	0.088 to 0.228
eGFR (mL/min/1.73 m^2^)	−0.010			0.66	
Heart rate (bpm)	−0.046			0.12	
DBP (mmHg)	0.008			0.90	
Troponin (ng/mL)	5.444			0.87	
Ferritin (ng/mL)	0.007			0.15	
LAD (mm)	0.126			0.19	

* *p* < 0.05, statistically significant. B = unstandardised coefficient; Beta = standardised coefficient. DBP: diastolic blood pressure; eGFR: estimated glomerular filtration rate; LVEF: left ventricular ejection fraction; LAD: left atrial diameter.

**Table 4:** Correlation of laboratory parameters with echocardiography findings. Correlation between RV-LS and ferritin, haemoglobin, and pulmonary acceleration time.

Parameter	N	Correlation Coefficient (r)	Standard Error (SE)	* p * -Value
Ferritin vs. RV-LS	99	0.21	-	0.04 *
Ferritin vs. pulmonary acceleration time (ms)	99	0.25	-	0.02 *
Haemoglobin vs. RV-LS	99	0.32	-	0.01 *

* *p* < 0.05, statistically significant. RV-LS: right ventricular longitudinal strain; SE: standard error.

## Data Availability

The original contributions presented in this study are included in the article/Supplementary Material. Further inquiries can be directed to the corresponding author.

## Supplementary Materials

The following supporting information can be downloaded at https://www.mdpi.com/article/10.3390/jcm14155188/s1. Figure S1: RV-LS vs. Haemoglobin; Figure S2: RV-LS vs Ferritin; Figure S3: RV-LS vs Pulmonary Acceleration.
