# Supplementary material for: Effect of Iron Deficiency on Right Ventricular Strain in Patients Diagnosed with Acute Heart Failure
Source: J Clin Med. 2025 Jul 22;14(15):5188. doi: 10.3390/jcm14155188 (PMC12347985; doi:10.3390/jcm14155188)
Supplement: Supplementary file 1 [file jcm-14-05188-s001.zip › jcm-3691932-supplementary.pdf]

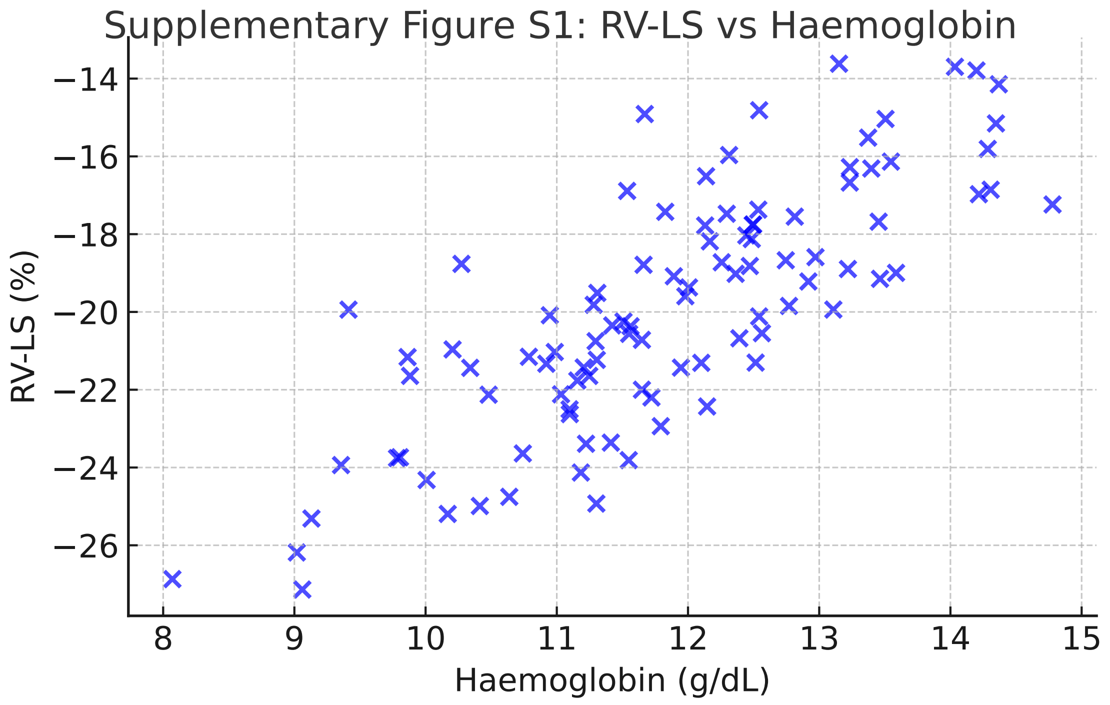
Supplementary Figure S1: RV-LS vs Haemoglobin
RV-LS (%)
−14
−16
−18
−20
−22
−24
−26
8
9
10
11
12
13
14
15
Haemoglobin (g/dL)

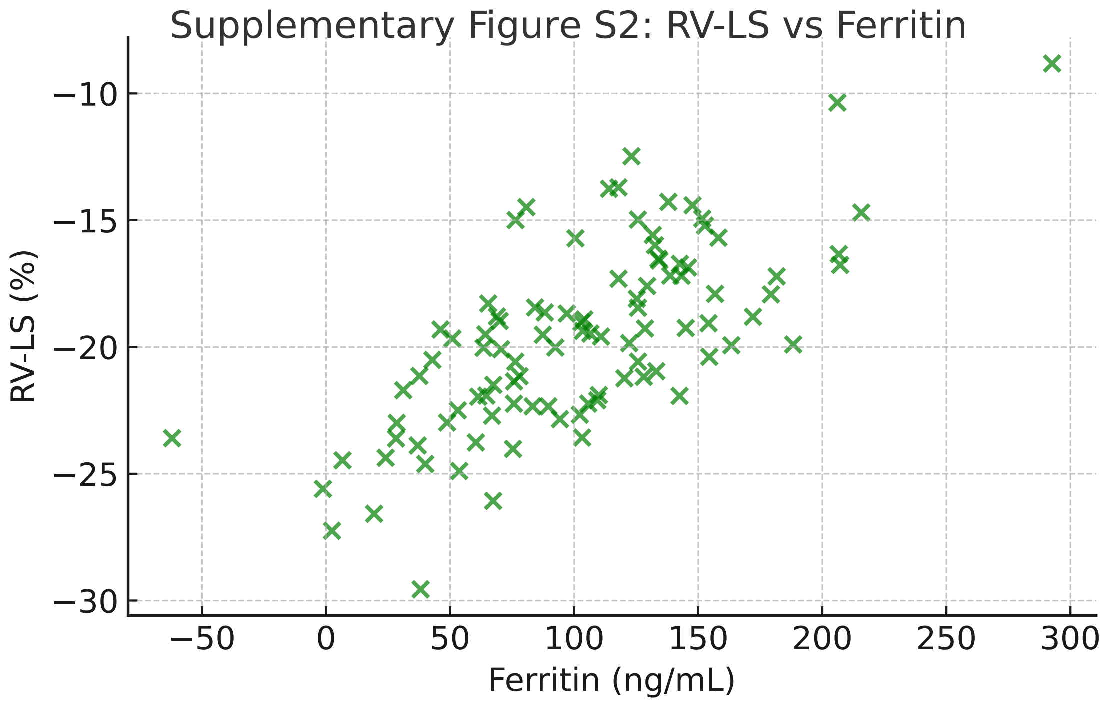
Supplementary Figure S2: RV-LS vs Ferritin
RV-LS (%)
−10
−15
−20
−25
−30
−50
0
50
100
150
200
250
300
Ferritin (ng/mL)

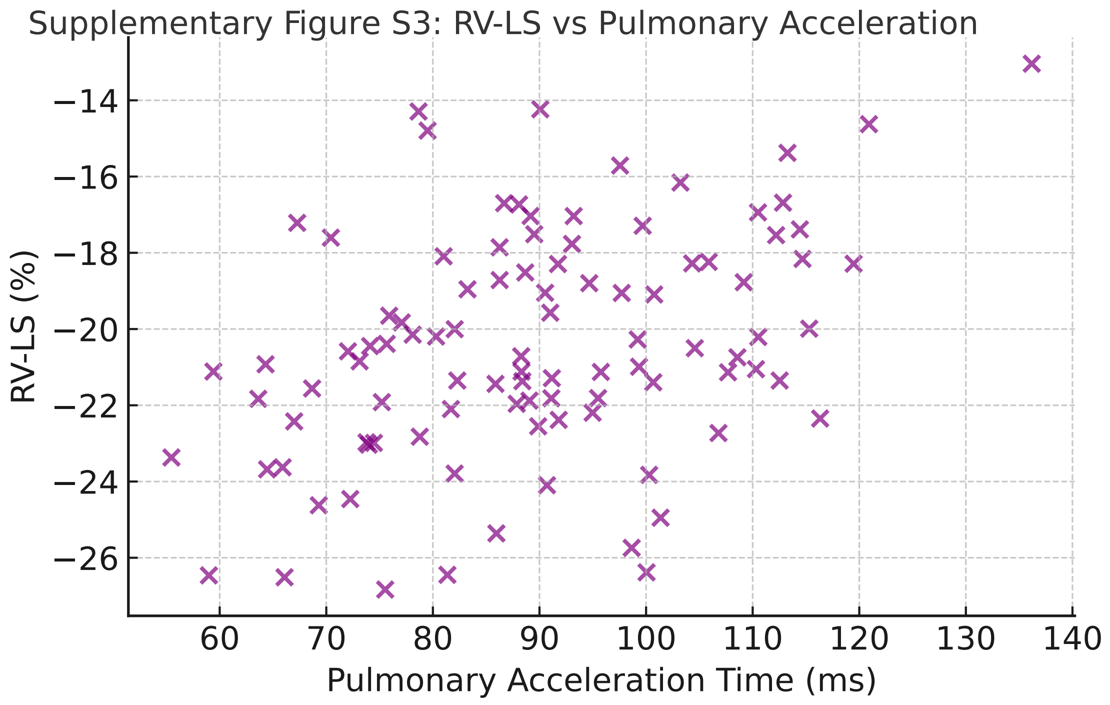
Supplementary Figure S3: RV-LS vs Pulmonary Acceleration
RV-LS (%)
−14
−16
−18
−20
−22
−24
−26
60
70
80
90
100
110
120
130
140
Pulmonary Acceleration Time (ms)
